# Supplementary material for: Aggressiveness in Italian Children with ADHD: MAOA Gene Polymorphism Involvement
Source: Diseases. 2024 Mar 31;12(4):70. doi: 10.3390/diseases12040070 (PMC11049508; doi:10.3390/diseases12040070)
Supplement: Supplementary file 1 [file diseases-12-00070-s001.zip › diseases-2912117-supplementary.pdf]

**Table S1. Descriptive statistics of Conner's Rating Scales completed by caregivers. Means (and Standard Deviations) of the ADHD subscales: (A) ADHD index, Inattention and Hyperactivity and (B) Defiance/Aggression in the total sample, boys and girls.**

**A**

| SUBSCALE | TOTAL SAMPLE (n=80) | BOYS (n=74) | GIRLS (n=6) |
|---|---|---|---|
| ADHD index | 75,017 (15,817) | 74,111 (15,773) | 90,250 (15,909) |
| Inattention | 70,682 (11,796) | 70,639 (12,102) | 71,200 ( 7,981) |
| Hyperactivity | 71,162 (13,276) | 70,344 (13,264) | 77,500 (12,629) |

**B**

| SUBSCALE | TOTAL SAMPLE (n=80) | BOYS (n=74) | GIRLS (n=6) |
|---|---|---|---|
| Defiance/Aggression (All patients) | 70,394 (23,685) | 69,820 (23,918) | 77,400 (21,640) |
| **SUBSCALE** | **TOTAL SAMPLE (n=32)** | **BOYS (n=28)** | **GIRLS (n=4)** |
| Defiance/Aggression (Aggressive patients) | 89,156 (20,453) | 90,071 (20,620) | 82,750 (21,640) |

*All the numbers are T scores*
